# Scenario-driven shifts in future Usutu and West Nile virus outbreak characteristics in the Netherlands

**DOI:** 10.1038/s41598-026-41926-0

**Published:** 2026-03-05

**Authors:** Mariken M. de Wit, Martha Dellar, Gertjan Geerling, Eline Boelee, Mart C. M. de Jong, Quirine A. ten Bosch

**Affiliations:** 1 Infectious Disease Epidemiology, Department of Animal Sciences, Wageningen University and Research, Wageningen, The Netherlands; 2 Deltares, Daltonlaan 600, Utrecht, 3584BK The Netherlands; 3 Department of Environmental Science, Radboud Institute for Biological and Environmental Sciences, Radboud University, Nijmegen, The Netherlands

**Keywords:** Climate sciences, Diseases, Ecology, Ecology, Environmental sciences

## Abstract

Mosquito-borne virus transmission is shaped by its ecological context, including land use, climate, and population dynamics. Future changes in these factors may therefore affect the risk and intensity of Usutu virus (USUV) and West Nile virus (WNV) outbreaks in the Netherlands. We compared a reference scenario to four future Shared Socio-economic Pathway scenarios developed for the Netherlands, which differed in land use, host distribution, mosquito distribution, and temperature. Temperature (between + 1.0 °C and + 1.7 °C) and mosquito abundance (between + 5% and + 10%) were predicted to increase during the transmission season across the scenarios. Scenario effects on hosts differed between species with the largest abundance increases predicted for WNV hosts. We found that outbreak size and growth rate were expected to increase in all future scenarios for both USUV and WNV. These effects were most pronounced early in the season and in scenarios characterised by a high temperature increase. Changes in outbreak risk differed between locations due to spatial variation in changes in host and vector abundance, but risk generally increased most in Southern and Eastern regions. Across a range of possible future scenarios, USUV and WNV outbreaks are expected to become larger, grow faster, and last longer. This is mostly driven by increased temperatures, highlighting the importance of climate mitigation measures to reduce disease outbreak risk and impact.

## Introduction

Transmission dynamics of mosquito-borne diseases are influenced by a complex interplay of ecological factors, including climate, land use, biodiversity, and population dynamics^1^. This ecological context shapes the interactions between the mosquitoes and hosts required for transmission. Therefore, ecological changes can affect the risk of outbreaks, persistence, and emergence of new arboviruses^2^. Urbanisation has been associated with increased *Aedes* mosquito density and virus transmission, such as for dengue fever^3^ as well as with increased *Culex pipiens* density^4^. Similarly, deforestation has been linked to habitat changes which may affect mosquito and host distributions, between-species contact rates, and movement, and could thereby increase outbreak risk as has been shown for a wide range of mosquito-borne diseases including malaria and Chikungunya^5–7^. One of the most studied environmental drivers of transmission is temperature. Temperature impacts a mosquito’s lifespan, biting frequency, and development, as well as viral replication rates within a mosquito, thereby enhancing transmission potential in multiple ways, including the length of the transmission season^8^. These ecological drivers, combined with social drivers like global travel and trade, have contributed to the geographic expansion of mosquito-borne diseases in recent decades^2,9^, and are expected to continue to do so^1,10^. Due to the diversity in mosquito and host species and local variation in their distribution, the exact impact of these changes is not straightforward to predict and varies by disease, species, and geographical area.

Two closely related arboviruses that have recently emerged in birds in Europe (including the Netherlands) are Usutu virus (USUV)^11^ and West Nile virus (WNV)^12^. USUV was first detected in Europe in 2001 following an observed increase in bird deaths in Austria^13^. It since spread to several European countries, including the Netherlands where it expanded from South to North leading to large blackbird population declines during the first three years after emergence^14,15^. The first large European outbreak of WNV occurred in Romania in 1996^16^. Recently, outbreaks have most frequently been reported in Italy and Greece including in humans^17^. Contrary to in the United States^18^, high WNV-associated bird mortality has not been observed in Europe. The first detection of WNV in both humans and birds in the Netherlands occurred in 2020^19^. While no human cases have been reported there since 2020, seroprevalence levels in birds suggest continued low-level circulation^15^. Both USUV and WNV are transmitted in a cycle between mosquitoes and birds, with *Culex pipiens* likely being the most important vector species in Europe^20^. The host ranges of USUV and WNV show a high degree of overlap, with at least 34 bird species in common across 11 orders^20^. However, while both viruses can infect humans, the impact on human health is larger for WNV as symptoms are more severe^11^. Another important difference is that USUV emergence in Europe has been associated with high Eurasian Blackbird (*Turdus merula*, hereafter: blackbird) mortality^13,15,21–23^, while such noticeable impacts on bird mortality have not been reported for WNV in Europe. The spread of WNV across Europe has been associated with land use, especially agricultural activity (e.g. livestock density, coverage of cropland)^24^, and climate, including spring and summer temperatures and precipitation^25,26^.

The Netherlands is a water-rich country, with two large rivers forming a delta, with high human and livestock population densities. Such urbanised delta ecosystems are particularly vulnerable to environmental changes, such as increased flooding risk, sea-level rise, and extreme climate events^27^. Dellar et al.^28^ developed national One Health scenarios for the Netherlands for 2050 based on the global Shared Socio-economic Pathways (SSPs). SSPs are global scenarios for future societal development, including factors such as demographics, economics, technology, governance, and the environment^29^, that can be combined with climate scenarios (Representative Concentration Pathways (RCPs)). The impact of these One Health scenarios has been quantified for land use^30^, distribution of several bird species^31^, and *Culex pipiens* abundance^32^ in the Netherlands. It is, however, not yet known how these changes in temperature and population distributions interact and thereby shape the risk of future USUV and WNV outbreaks. Quantifying the impact of future scenarios on arbovirus disease risk helps in understanding the possible consequences of policy choices and allows for an informed decision-making process. Long-term outbreak predictions can also facilitate preparedness planning.

We leveraged highly comprehensive and detailed One Health scenarios to assess future scenarios of USUV and WNV outbreaks in the Netherlands. We calculated several measures of outbreak risk and intensity and compared these between a reference scenario and four SSPs. These measures were calculated over space and time and stratified by land use class and province.

## Methods

Local transmission is governed by interactions between hosts and mosquitoes and is sensitive to temperature. Therefore, host abundance, mosquito abundance, and temperature were obtained for the different scenarios for the Netherlands. To calculate local differences in outbreak risk measures, the country was divided into 5 × 5 km grid cells. Outbreak risk measures were calculated daily for each grid cell in the Netherlands from April to November. The reference scenario represents the current situation, while the future scenarios represent a multi-year average centered around 2050. Scenario impacts were quantified using several key epidemiological metrics. Local outbreak risk was determined by calculating the basic reproduction number (R_0_), which determines the average number of secondary cases resulting from one average infectious individual in a fully susceptible population. The explosiveness of an outbreak was quantified by calculating the epidemic doubling time (i.e. the time it takes for the outbreak to double in size in a fully susceptible population).

### Model overview

**Fig. 1:**
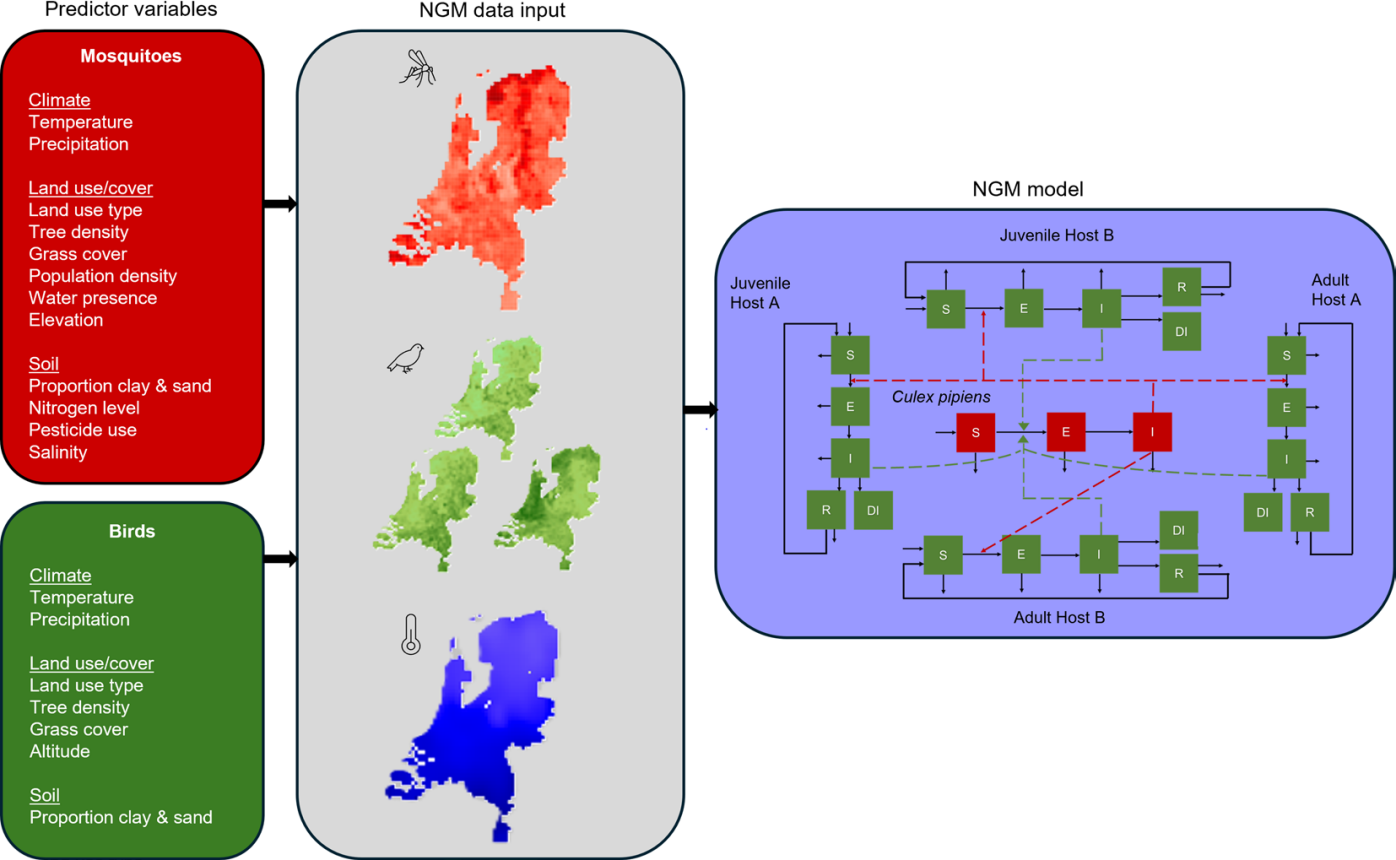
Model and data overview. Environmental predictor data is combined with bird and mosquito occurrence data to create spatial (for birds) and spatiotemporal (for mosquitoes) abundance maps, see^31,32^. The country is divided into 5 × 5 km grid cells, which vary with respect to bird and mosquito abundance as well as temperature. For USUV, the host population consists of blackbirds and an additional reservoir population. For WNV, the host population consists of a high and a low infectious population represented by house sparrows (high infectiousness) and mallards (low infectiousness). All bird populations were split into juveniles and adults. R_0_ and generation time related measures are calculated at grid cell level based on the NGM. In the NGM model, dark arrows represent transitions between infection states, while dashed arrows represent the link to the source of transmission. S = susceptible, E = exposed, I = infectious, R = recovered, DI = dead from infection. Non-connected outward pointing arrows represent natural death of the animal. Non-connected inward pointing arrows represent births.

We developed a next generation matrix (NGM) to model USUV and WNV transmission between *Culex pipiens* mosquitoes and bird host populations (Fig. 1). Dead-end hosts, such as humans and horses, were not explicitly included in the model as they do not contribute to onwards transmission. In this model, virus transmission occurred within grid cells, assuming a homogeneous distribution of birds and mosquitoes within these cells. Birds can get infected when they get bitten by an infectious mosquito, while mosquitoes can get infected when they bite an infectious bird. We did not include connections between cells. The mosquito mortality rate, extrinsic incubation period, and biting rate were assumed to be temperature dependent. Parameter values were obtained from literature for both USUV and WNV (Tables 1 and 2). Because exact population size estimates are lacking for relevant hosts and mosquitoes, we used an abundance scaling parameter to convert the host abundance to the same scale as the mosquito abundance, such that their ratio reflects the vector-to-host ratio. R_0_ was calculated from this NGM for each location and day (Supplementary Material A). Each element in the NGM, *k*_*ij*_, represents the expected number of infections of type *i* caused by an individual of type *j*. The dominant eigenvalue of this matrix equals R_0_.

The host population for USUV consisted of two groups: blackbirds and an additional reservoir population. Blackbirds were included as a host species because high prevalence and high USUV-related mortality has been observed in this population^21,22,33^. An additional animal reservoir population was included, because it has been shown that this population played a large role in USUV transmission in earlier outbreaks in the Netherlands^34^. This population consists of multiple (bird) species which are able to transmit the virus to mosquitoes and contribute to ecological persistence of the virus. The host population for WNV also consisted of two host groups which differed with respect to their infectiousness and mortality rate. The ‘high infectiousness’ population was represented by House Sparrows (*Passer domesticus*, hereafter: house sparrow), while the ‘low infectiousness’ population was represented by Mallards (*Anas platyrhynchos*, hereafter: mallard)^35^. Mortality rate due to WNV infection was higher in house sparrows than in mallards^35^. Further information on this choice of species for WNV is presented in Supplement A. Due to differences in survival rates across life stages, we divided all bird populations into juveniles (from fledging until the next breeding season) and adults (birds older than one year). We assumed susceptibility and infection mortality to be the same for both age groups^36,37^.

*Culex pipiens* mosquitoes bite a wide range of hosts^38^, including hosts unable to further transmit the virus (i.e. non-competent hosts). To account for the presence of non-competent hosts, we calculated the proportion of mosquito bites taken on competent hosts. We assumed that the modelled bird populations represent all competent hosts and that the total number of (competent and non-competent) hosts is the same in each location. We assumed that the proportion of bites on competent hosts is proportional to the relative abundance of competent host species in a grid. To reflect this, we calculated this proportion as the relative abundance compared to the cell where abundance was highest, with the grid cell with the highest competent host abundance set to 100%.

**Table 1:** Parameters and their values used in the NGM for USUV. T indicates temperature, t indicates time. HDI = highest density interval.

Parameter	Description	Value	Source
Blackbirds			
δ_b_(t)	Daily number of births	1 May – 15 June: 0.056 per bird Else: 0	Value: see supplementary material. Timing^39^:
µ_JB_	Mortality rate, juvenile	0.0059/day	^39^
µ_AB_	Mortality rate, adult	0.0011/day	^40^
p_bm_	Transmission probability per bite, mosquito to blackbird	0.88	^41^
ε	Intrinsic incubation rate	0.67/day	^42,43^
γ	Recovery rate	0.25/day	^44^
ν_b_	Disease-induced mortality rate	0.69/day (95% HDI 0.49–0.88)	Posterior distribution obtained from^34^
**Additional reservoir population**			
δ_r_(t)	Daily number of births	1 May – 15 June: Variable, depending on value for mortality Else: 0	Value: see supplementary material. Timing^39^:
µ_JR_	Mortality rate, juvenile	0.0059/day	Set equal to blackbird juvenile
µ_AR_	Mortality rate, adult	4.7*10^− 4^/day (95%HDI 2.1*10^− 4^ − 2.5*10^− 3^)	Posterior distribution obtained from^34^
p_bm_	Transmission probability per bite, mosquito to reservoir host	0.88	^41^
ε	Intrinsic incubation rate	0.67/day	^42,43^
γ	Recovery rate	0.25/day	^44^
ν_r_	Disease-induced mortality rate	0	^34^
ω	Relative biting frequency	42.0 (95% HDI 32.3–49.8)	Posterior distribution obtained from^34^
***Culex pipiens***			
µ_m_(T)	Mortality rate [daily]	For T < 33 ^◦^C: 1/(−4.86*T + 169.8), else 0.17	^45^ fitted to data from^46–48^
b(T)	Biting rate [daily]	0.344/(1 + 1.231*exp(−0.184*(T-20)))	^43^ fitted to data from^49^
q	Proportion of bites on competent host	Variable	Calculated, see supplementary material.
p_mb_	Transmission probability per bite, blackbird & reservoir population to mosquito	0.79 (95% HDI 0.43–1.0.43.0)	Posterior distribution obtained from^34^
u(T)	Extrinsic incubation rate [daily]	For T > 13 ^◦^C: 7.38*10^− 5^ * T * (T – 11.4)(45.2 – T)^1/2^, else 0	^45^ fitted to data from^50,51^
σ(t)	Rate of entering diapause	After 1 September: 0.05. Else: 0	^52^
α	Abundance scaling parameter	0.11 (95%HDI 0.05–0.14)	Posterior distribution obtained from^34^

**Table 2:** Parameters and their values used in the NGM for WNV. T indicates temperature, t indicates time. HDI = highest density interval.

Parameter	Description	Value	Source
House Sparrow			
δ_s_(t)	Daily number of births	1 May – 15 June: 0.020 per bird Else: 0	Value: see supplementary material. Timing^39^:
µ_JS_	Mortality rate, juvenile	0.002/day	^53^
µ_AS_	Mortality rate, adult	0.0015/day	^53^
p_bm_	Transmission probability per bite, mosquito to house sparrow	0.88	^41^
ε	Intrinsic incubation rate	1/day	^35^
γ	Recovery rate	0.22/day	^35^
ν_S_	Disease-induced mortality rate	0.21/day	^35^
**Mallard**			
δ_m_(t)	Daily number of births	1 May – 15 June: 0.016 per bird Else: 0	Value: see supplementary material. Timing^39^:
µ_JM_	Mortality rate, juvenile	0.0018/day	^54^
µ_AM_	Mortality rate, adult	0.0013/day	^54^
p_bm_	Transmission probability per bite, mosquito to mallard	0.88	^41^
ε	Intrinsic incubation rate	1/day	^35^
γ	Recovery rate	0.25/day	^35^
ν_m_	Disease-induced mortality rate	0	^35^
ω	Relative biting frequency	1	Assumed
***Culex pipiens***			
p_ms_	Transmission probability per bite, house sparrow to mosquito	0.98	Viremia levels^35^: Translation viremia to infectiousness^55^: Calculation: supplementary material
p_mm_	Transmission probability per bite, mallard to mosquito	0.43	Viremia levels^35^: Translation viremia to infectiousness^55^: Calculation: supplementary material

All other *Culex pipiens* parameters were identical to those shown in Table 1.

### Development of future change scenarios

**Fig. 2:**
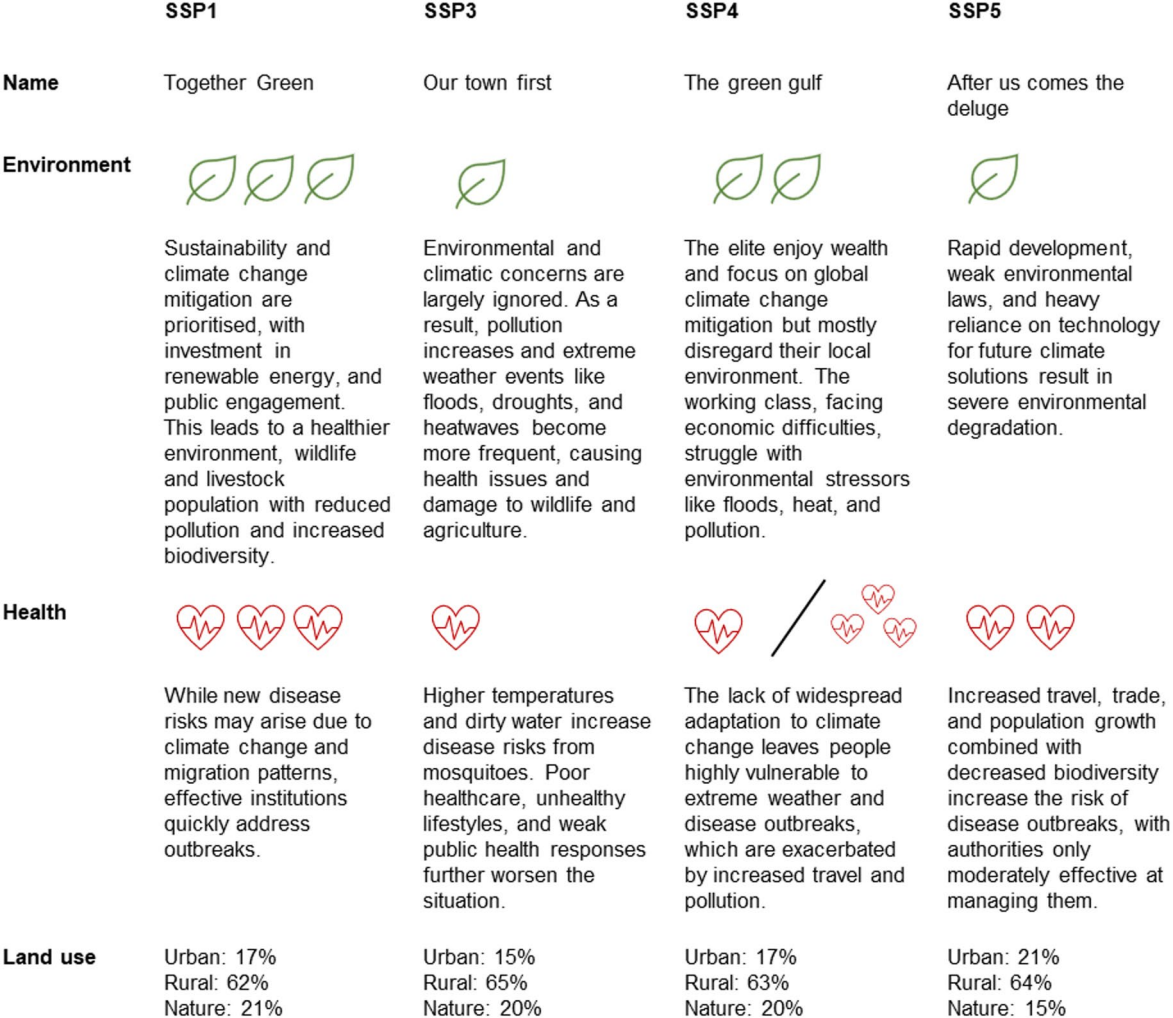
Qualitative description of future One Health change scenarios for the Netherlands. A more detailed description is available in Dellar et al.^28^.

We compared USUV and WNV transmission potential, over time and space during the transmission season, under a reference scenario and four future scenarios for the Netherlands. The reference scenario represents the current situation, while the future scenarios are based on the Dutch One Health scenarios derived from the global Shared Socio-economic Pathways (SSP) by Dellar et al^28^.. The scenarios were developed by combining existing scenarios (including global and European SSPs), planned (inter)national policies, (grey) literature, and stakeholder consultations. A total of four scenarios were developed: SSP 1 (“Together green”), SSP 3 (“Our town first”), SSP 4 (“The green gulf”), SSP5 (“After us comes the deluge”) (Fig. 2).

These SSP scenarios were used to derive future mosquito and bird abundance and distribution. Impacts of scenarios were quantified based on changes in a wide range of factors, including human population, vegetative cover, water quality, and climate. These changes were derived from scenario-specific land use maps^30^, qualitative SSP scenarios^28^ and climate scenarios from the Royal Netherlands Meteorological Institute (KNMI)^56^. Scenario-specific blackbird, house sparrow, mallard, and *Cx. pipiens* abundance maps were developed that incorporate these changes (^31^ for birds^32^, for *Cx. pipiens*). Bird distributions were created for the breeding season using Random Forest models fitted to point count data from the Netherlands (Meetnet Urbane Soorten^57^, Meetnet Agrarische Soorten^58^ and Common Bird Census^59^ from Sovon Vogelonderzoek Nederland (Dutch Centre for Field Ornithology)) and France (Common Bird Monitoring Scheme^60^), using a set of environmental and climatic predictors. Data from France were included because the current French climate is deemed similar to the future Dutch climate. Mosquito distributions were created using a similar approach where data on female mosquito trap counts was used from the National Mosquito Survey 2010–2013^61^ and the MODIRISK project^62^.

Future climate data was taken from scenarios developed by the KNMI for the period 2036–2065^56^. This consists of six scenarios: low, medium, and high greenhouse gas emissions, each with a wet and dry variant. The different emissions levels are approximately equivalent to RCPs (Representative Concentration Pathways) 2.6, 4.5 and 8.5 respectively. Each scenario is the result of an 8-model ensemble and uses 1991–2020 as a reference period. The scenarios are on a 12 km grid, which we converted to a 5 km grid using bilinear interpolation. For our analysis, SSP 1 was paired with the low emissions scenarios, SSP 4 was paired with the medium emissions scenario, and SSPs 3 & 5 were paired with the high emissions scenarios. To calculate temperature-dependent parameters, we averaged over the 30 years, the 8 ensembles and the wet and dry variants. Future scenarios thereby represent the average predicted temperature in 2050. The reference scenario was defined as the average across the KNMI reference period of 1991–2020.

Apart from the temperature-dependent parameters, parameter values remained the same across scenarios. Initiation and end of diapause was also kept equal to the reference scenario, because these processes, at least partially, depend on day length which does not change in future^63^. Due to a lack of information on the future distribution of the non-competent host population, the proportion of bites on competent hosts averaged across all grid cells was also kept equal to the reference scenario. Locally, the proportion of bites on competent hosts could still differ between scenarios.

### Scenario-specific abundance and parameters

**Fig. 3:**
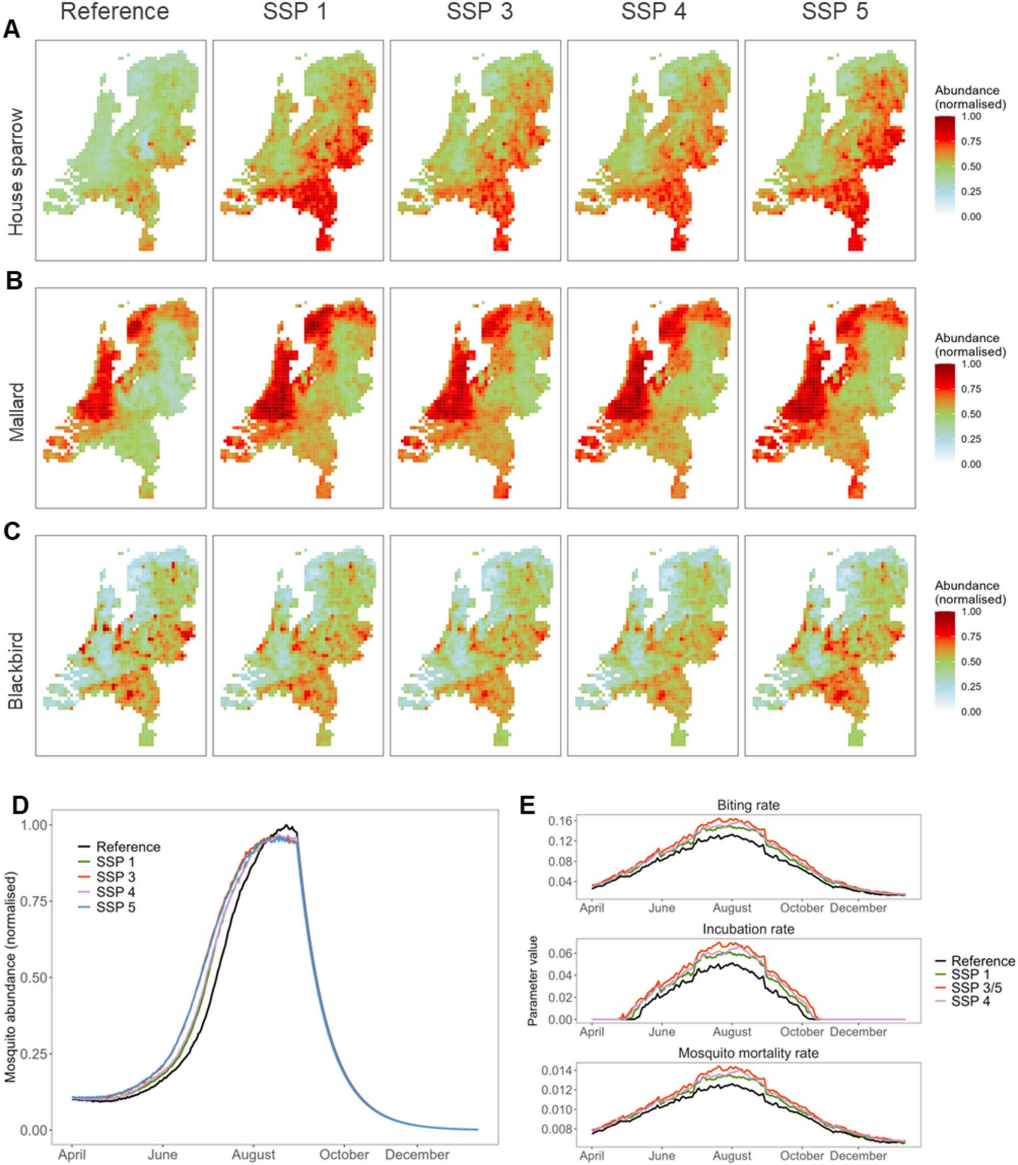
Present and future (year 2050) estimates of breeding season abundance for (**A**) House Sparrows, (**B**) Mallards, and (**C**) Blackbirds, (**D**) Cx pipiens abundance, (**E**) temperature-dependent parameters. Spatial visualizations of mosquito abundance and temperature are available in the supplement.

Abundance, temperature, and therefore all temperature-dependent parameters, differed between scenarios. Overall, blackbird abundance remained relatively similar across all scenarios, while both mallard and house sparrow abundance increased (Fig. 3A-C, supplementary Fig. 1). The largest increase compared to the reference scenario was observed in SSP1 for house sparrows. On a local level, house sparrow and mallard abundance increased almost everywhere, while the limited changes in blackbird abundance were a combination of local increases and decreases (Supplementary Fig. 1). Across all scenarios, mosquito abundance increased between 4.7% (95%CI 3.3–6.1) in SSP1 and 9.6% (95%CI 8.3–11.0) in SSP3. This increase was most prominent early in the year, while peak abundance was somewhat reduced compared to the reference scenario (Fig. 3D, spatial results: supplementary Fig. 2). Temperature-dependent parameters were highest for SSP3 and 5 as these scenarios are associated with the highest temperatures (Fig. 3E). Average temperature increases across the period April-November ranged from + 1.0 °C (95%CI 1.00–1.05.00.05) in SSP1 to + 1.7 °C (95%CI 1.66–1.71) in SSP3 and 5 (supplementary Fig. 3).

### Analyses

Scenarios were compared based on three important metrics of potential pathogen transmission: the basic reproduction number *R*_*0*_, the generation time *T*, and the epidemic growth rate *r*.

### Basic reproduction number

Basic reproduction numbers (R_0_) were calculated using the Next Generation Matrix approach^64^ (supplementary material A). R_0_ indicates the average number of secondary cases resulting from one average infectious individual in a fully susceptible population. Uncertainty in parameter estimates was incorporated in R_0_ estimates by sampling 100 parameter sets from the posterior distributions (Table 1) taken from^34^.

### Generation time

The generation time *(T*) is defined as the time interval between successive infections in hosts. We derived the mean generation time for both viruses as the sum of four sequential steps in the transmission cycle, adapted from^65^.

- Intrinsic incubation period.

This period was defined as the time between a host getting infected and becoming infectious. This was calculated by taking the inverse of the intrinsic incubation rate (ε) (Tables 1 and 2). When estimates varied between host species, we used the average.

- Host-to-mosquito transmission period.

This period was defined as the time between a host becoming infectious and a susceptible mosquito getting infected from biting this infectious host. Assuming a constant infectiousness and hosts being bitten at a constant rate during the infectious period, a mosquito infection occurs, on average, halfway through the infectious period. We therefore defined the mean host-to-mosquito transmission period as half of the host infectious period. The host infectious period was calculated as the sum of the inverse of the recovery rate (γ), disease-induced mortality rate (ν), and natural mortality rate (µ) (Tables 1 and 2). Again, when estimates varied between host species, we used the average.

- Extrinsic incubation period.

This period represents the time between a mosquito getting infected and becoming infectious. This was calculated by taking the inverse of the extrinsic incubation rate (u(T)) (Tables 1 and 2).

- Mosquito-to-host transmission period.

This period was defined as the time between a mosquito becoming infectious and transmitting the infection to a susceptible host. When we assume a constant infectiousness and mortality rate, transmission occurs, on average, halfway through the infectious period (i.e. halfway through a mosquito’s lifespan). We therefore defined the mean mosquito-to-host transmission period as half a mosquito’s lifespan, calculated by taking the inverse of the mortality rate (µ_m_(T)) (Table 1).

The number of generations that occur in a year depends not only on the generation time, but also on the duration of the transmission season. Both a reduced generation time and/or a longer transmission season can lead to more generations per year. We defined the duration of the transmission season as the number of days during which R_0_ > 1.

### Epidemic growth rate

While the reproduction number provides information on the number of secondary cases arising from an infectious individual, it does not provide information about how quickly these secondary cases occur. The epidemic growth rate is defined as the rate of change in the (log-transformed) number of new cases per unit of time and provides insight into the explosiveness of an epidemic. The relationship between the epidemic growth rate and R_0_ depends on the distribution of the generation interval^66^. Assuming that all generation intervals are equal to the mean generation interval, the relationship between R_0_ and the growth rate can be expressed as

1 $${R}_{0}={e}^{rT}$$

where *r* is the epidemic growth rate, and *T* is the mean generation interval. The growth rate is directly related to, the more intuitive, epidemic doubling time (TD) which represents the time it takes for the number of cases to double. This can be expressed as

2 $$\mathrm{T}\mathrm{D}=\frac{\mathrm{ln}\left(2\right)}{r}$$

### Sensitivity analyses

We conducted a sensitivity analysis to assess the implications of our choice of host species for WNV. Here, we assumed the host population to be uniformly distributed across the country. The average proportion of bites on competent hosts was kept equal to the main analysis.

## Results

### Scenario comparison across season

**Fig. 4:**
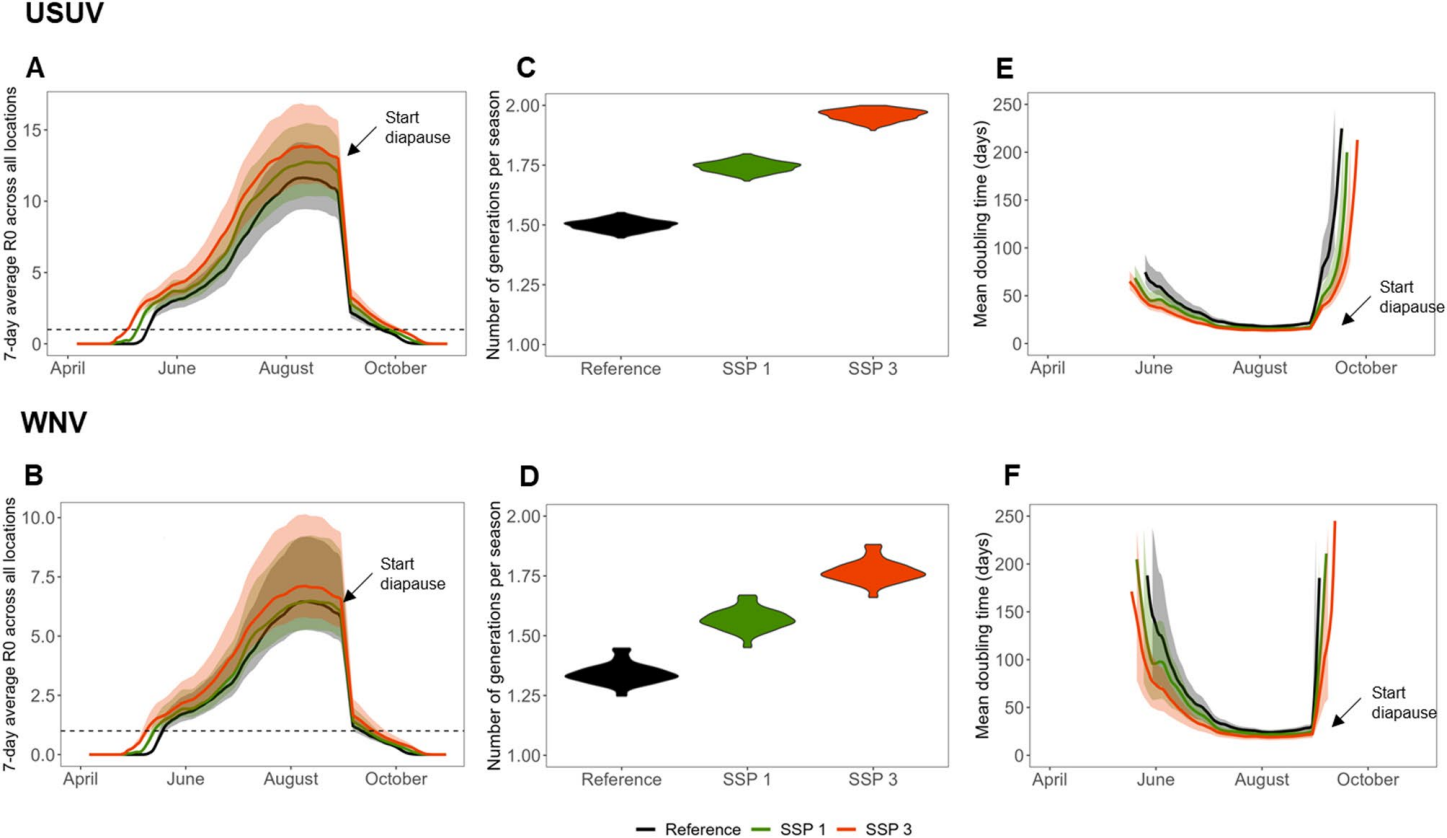
Comparisons of basic reproduction number (**A-B**), number of generations per season (**C-D**), and epidemic doubling time (**E-F**) between the reference scenario and SSP1 and SSP3. Shaded areas in A, B, E, and F represent the 95%CI based on variation in parameter estimates sampled from posterior distributions. Plots A, C, and E relate to USUV, while B, D, and F relate to WNV. Note that the y-axis scales differ between figures A and B.

Four future scenarios were compared to a reference scenario across a range of outbreak risk measures, with scenarios differing in land use, host distribution, mosquito distribution, and temperature. All future scenarios were characterised by a longer transmission season (up to 17% longer) and higher R_0_ for both pathogens (Fig. 4A-B) when compared to the reference scenario. The average R_0_ was highest in SSP3 and lowest in SSP1 for both USUV (reference: 4.0 (95%CI 3.2–4.9), SSP3: 5.3 (95%CI 4.3–6.5), SSP1: 4.7 (95%CI 3.8–5.7)), and WNV (reference: 2.2 (95%CI 1.8–3.2), SSP3: 2.7 (95%CI 2.2–3.9), SSP1: 2.4 (95%CI 1.9–3.4)). Therefore, only results from these two extreme scenarios are presented in the main text (for other scenarios, see Supplementary Material B). R_0_ surpassed 1 earlier in the season in SSP1 (USUV: 7 days, WNV: 3 days) and even more so in SSP3 (USUV: 16 days, WNV: 9 days). Additionally, R_0_ also peaked at higher values, most notably for USUV (USUV reference: 11.7 (95%CI 9.5–14.3), SSP3: 14.0 (95%CI 11.4–17.0), SSP1: 12.8 (95%CI 10.4–15.6)) & WNV reference: 6.5 (95%CI 5.3–9.2), SSP3: 7.2 (95%CI 5.8–10.2), SSP1: 6.5 (95%CI 5.3–9.3)). The proportion of locations with R_0_ > 1 also increased earlier in the season in the future scenarios (supplementary Fig. 4 C & 5 C). Differences in the decline of R_0_ during September were small, as we assumed that diapause initiation remained constant between scenarios.

We estimated a reduction in the generation time across all scenarios (supplementary Fig. 4E & 5E), due to higher temperatures. A reduced generation time and increased season length (i.e. period during which R_0_ > 1), results in more generations per season. Again, this effect was strongest in SSP3 (Fig. 4C-D). The number of generations changed from 1.5 (95%CI 1.5–1.5) in the reference scenario to 1.7 (95%CI 1.7–1.8) in SSP1 and 2.0 (95%CI 1.9–2.0.9.0) in SSP3 for USUV and from 1.3 (95%CI 1.3–1.4) in the reference scenario to 1.6 (95%CI 1.5–1.7) in SSP1 and 1.8 (95%CI 1.7–1.9) in SSP3 for WNV. Higher R_0_ combined with shorter generation time make outbreaks more explosive as the growth rate increases (supplementary Fig. 4D & 5D). Especially early in the transmission season, we observed shorter epidemic doubling times. In June, doubling times changed from 42 days (95%CI 28–61) in the reference scenario to 34 (95%CI 25–49) in SSP1 and 28 (95%CI 20–39) in SSP3 for USUV and 75 days (95%CI 43–129) in the reference scenario to 63 (95%CI 38–107) in SSP1 and 46 (95%CI 30–74) in SSP3 for WNV (Fig. 4E-F).

**Fig. 5:**
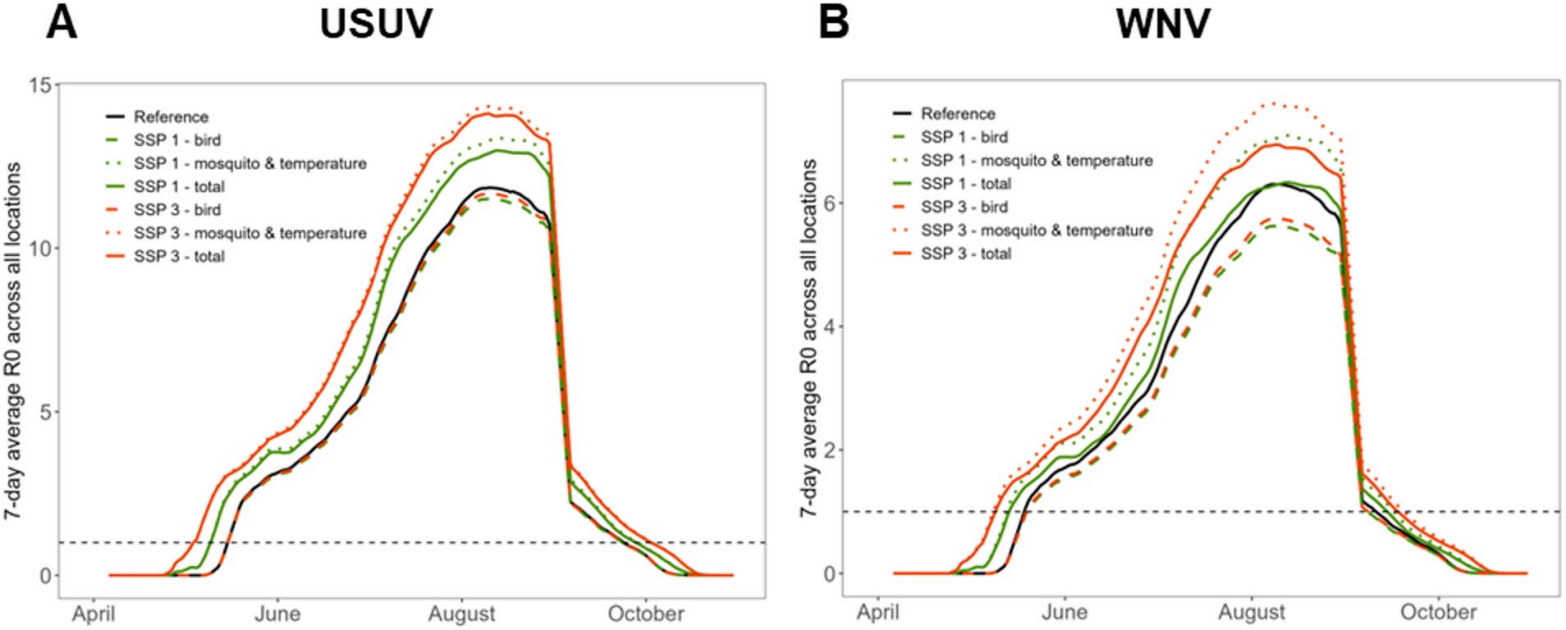
Comparison of R_0_ over time between reference and split-scenarios for USUV (**A**) and WNV (**B**). ‘Bird’ scenarios represent scenarios where only the bird abundance changes, while temperature and mosquito abundance remained equal to the reference scenario. ‘Mosquito & temperature’ scenarios represent scenarios where only the temperature and mosquito abundance change. ‘Total’ scenarios represent the standard scenarios where are elements change. Note that the y-axis scales differ between figures A and B.

To explore the contribution of changes in temperature and mosquito abundance compared to changes in bird abundance, we separated these effects for SSP1 and SSP3. We found that higher temperature and mosquito abundance were the most important drivers of increased R_0_ (Fig. 5).

### Scenario comparison across space

**Fig. 6:**
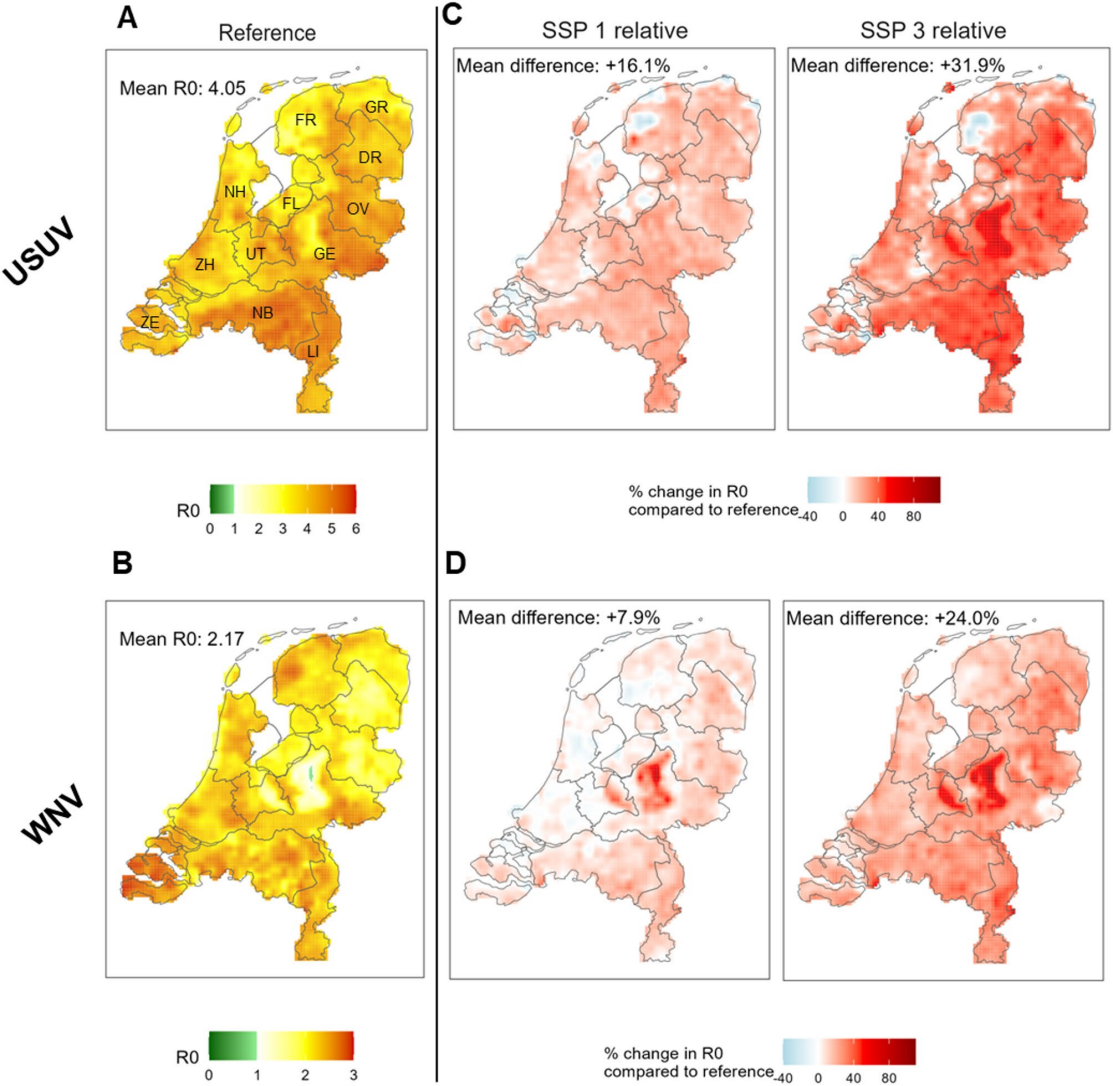
Map of basic reproduction number per grid cell for the reference scenario (**A-B**) and the change in the reproduction number between the scenarios and the reference (**C-D**). Note that scales differ between figures A and B. Abbreviations in figure A indicate province names: FR – Friesland, GR - Groningen, DR - Drenthe, OV – Overijssel, FL – Flevoland, GE – Gelderland, UT – Utrecht, NH – Noord-Holland, ZH – Zuid-Holland, ZE – Zeeland, NB – Noord-Brabant, LI – Limburg.

In the reference scenario, USUV R_0_ was highest in the South-Eastern region (Fig. 6A, provinces of Noord-Brabant (R_0_ 4.8, 95%CI 3.2–5.4), Limburg (R_0_ 4.7, 95%CI 3.9–5.3), and Gelderland (R_0_ 4.4, 95%CI 2.8–5.5)). This is mainly driven by high blackbird abundance in this region (Fig. 3). This is also the area where R_0_ increased most in both SSP1 and SSP3 for USUV (Fig. 6C, supplementary Fig. 6). We found high correlation between changes in USUV R_0_ for SSP1 and SSP3 (Pearson’s r 0.97) calculated at local level (i.e. 5 × 5 km).

WNV R_0_ was highest in the South in the reference scenario (Fig. 6B, highest value observed in the province of Zeeland (R_0_ 2.6, 95%CI 2.1–2.9)). For WNV, R_0_ increased most in the region where this was lowest in the reference scenario, which is currently a national park (Fig. 6D, supplementary Fig. 7). This coincides with the highest increase in (competent) bird abundance (supplementary Fig. 1). Whilst WNV R_0_ increased at the national level, this was not the case for all regions in SSP1. In the Northern and Western regions (provinces of Friesland, Utrecht, Noord-Holland, and Zuid-Holland), R_0_ remained stable. We found high correlation between changes in WNV R_0_ for SSP1 and SSP3 (Pearson’s r 0.91) calculated at local level (i.e. 5 × 5 km). As the mosquito population and temperature are the same for both USUV and WNV, spatial difference between both diseases are driven by variation in host abundance.

**Fig. 7:**
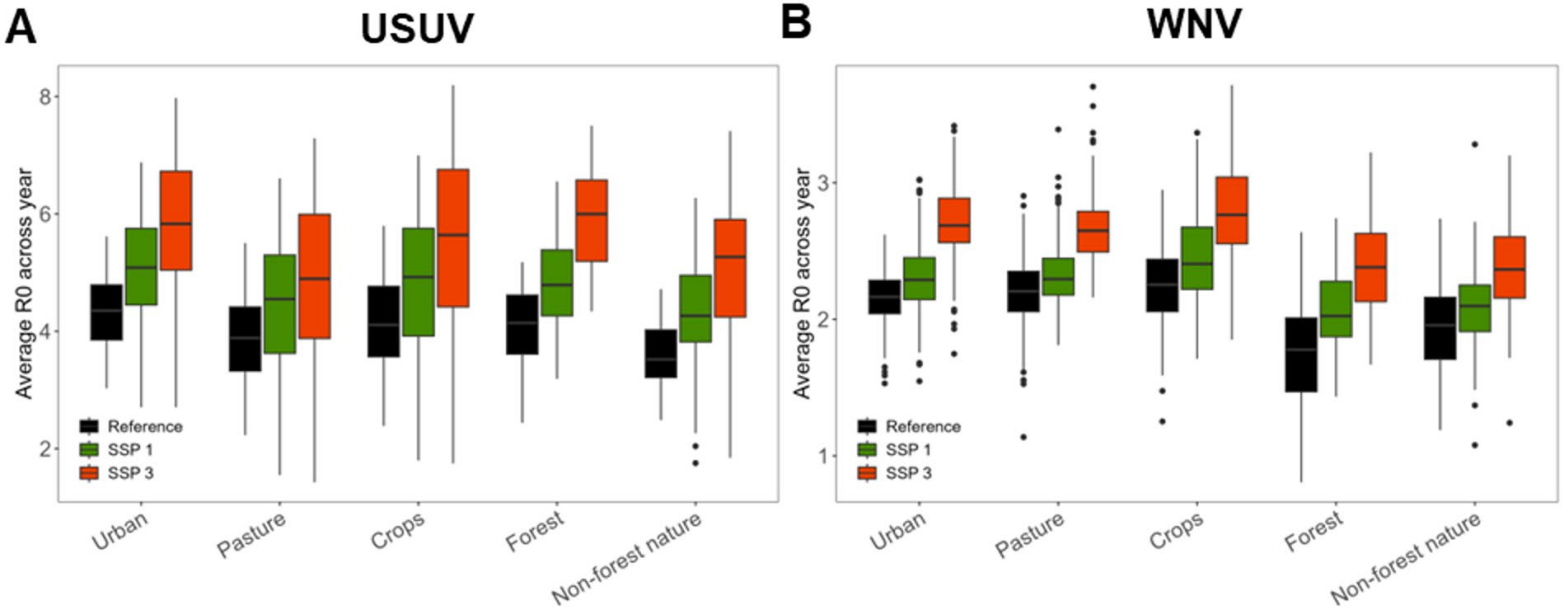
R_0_ averaged across the year per land use class for each main SSP scenario for (**A**) USUV and (**B**) WNV.

We also compared R_0_ values between land use classes, which were split into five categories: urban, pasture, crops, forest, and non-forest nature. While differences between land use classes could be observed in bird and mosquito abundance (supplementary Fig. 8), differences in R_0_ values were small (Fig. 7), with non-forest nature and forest being lowest for USUV and WNV respectively. The land use class associated with the highest R_0_ varied between provinces (supplementary Fig. 9, supplementary Fig. 10). Relative increases in R_0_ were similar across land use classes. For WNV, these ranged from 1.04 times in pasture to 1.14 times in forest for SSP1 and from 1.20 times in pasture to 1.34 times in forest for SSP3. For USUV, relative increases ranged from 1.16 times in forest to 1.21 times in non-forest nature for SSP1 and from 1.26 times in pasture to 1.50 times in non-forest nature for SSP3.

### Comparison between USUV and WNV

While R_0_ increased across all future scenarios for both USUV and WNV, there were local differences between these pathogens. We calculated correlations between these pathogens’ current R_0_ values and between their predicted change in future scenarios. In the reference scenario, we found low correlation between R_0_ values for USUV and WNV on a local level (i.e. 5 × 5 km) (Pearson’s r 0.22) and on a provincial level (Pearson’s r 0.30). Correlation between changes in R_0_ for both pathogens was higher on both a local level (Pearson’s r SSP1 0.53, SSP3 0.73) and a provincial level (Pearson’s r SSP1 0.51, SSP3 0.46).

### Sensitivity analyses WNV host distribution

To assess the implications of our choice of host species for WNV, we included a sensitivity analysis where we assumed mallards and house sparrows were uniformly distributed across the country. Mean R_0_ values were similar across scenarios (Supplementary Table 1), indicating that assumptions about host distribution do not impact the national-level effect of the scenarios on R_0_. On a more local level, spatial heterogeneity in the impact of scenarios was naturally reduced (supplementary Fig. 11).

## Discussion

Environmental changes can have important consequences for mosquito-borne disease risk. We found that the basic reproduction number increased in all future One Health scenarios developed for the Netherlands for both USUV and WNV, compared to the current situation. Additionally, the duration of the transmission season was projected to be longer, potentially leading to larger outbreaks as the number of generations per season increased. We estimated a reduction in the epidemic doubling time, especially early in the season, which makes outbreaks spread more rapidly. The increase in outbreak risk was most pronounced in SSP3 (‘Our town first’), which is characterised by a high increase in temperature and mosquito abundance. Overall increase in outbreak risk was largely driven by increased temperature and mosquito abundance. While transmission risk was predicted to increase at the national level in all scenarios, local predicted reductions in risk were observed in some locations in the North and West of the country, especially for SSP1. Spatial variation in risk increase was mostly due to spatial variation in changes in mosquito and bird abundance as temperature changes were more homogeneously distributed across the country. No clear differences were observed between land use classes. There was little correlation in current risk between USUV and WNV. The sensitivity analysis regarding the choice (and therefore distribution) of host populations for WNV showed that while local estimates were sensitive to these distributions, national trends of R_0_ between scenarios were robust to these.

Experimental studies suggest that WNV transmission will peak at average daily temperatures around 24.5 °C^45,67^. As mean daily temperatures of 24.5 °C were not reached even in SSP3, transmission was projected to increase in all scenarios and continued temperature rise is likely to further increase virus transmission. A European-wide study that modelled WNV risk for different climate change scenarios found an up to 3.5 times increase in population at risk of WNV infection in 2040–2060 compared to 2000–2020, with the largest risk increase observed in Western Europe^68^. Similar to our study, they observed the smallest increased risk for the low emissions scenario (RCP 2.6) and the largest increase in the high emissions scenario (RCP 8.5). However, this study did not incorporate land use changes and future host and vector abundance estimates. Secondly, this study focused on WNV geographical expansion, using a binary indicator of infection risk, instead of a continuous value such as R_0_.

We did not observe clear differences in transmission risk between land use classes (i.e. urban, pasture, crops, forest, non-forest nature). Land use explained about 37% of variation in abundance in all three bird species (i.e. blackbird, house sparrow, mallard)^31^ and almost 50% of variation in *Cx pipiens* abundance^32^. However, while species abundance differed between land use classes, the effect of land use varied between species. Their combined association with transmission risk was therefore reduced. This effect may be different in regions with different dominant host species. The choice of grid size may also affect the lack of observed variation in transmission risk between land use classes, as adult mosquito abundance has been found to vary locally between park and residential spaces within urban regions^69^. This highly local variation can also have an impact on transmission risk. While we assume that increasing mosquito abundance leads to increased transmission risk on the modelled scale (5 × 5 km), this impact could differ if mosquito and bird distribution is highly heterogeneous. For example, it has been suggested that mosquito numbers may increase locally, as a consequence of displacement due to drought. If this coincides with high bird density areas it could increase mosquito-bird contact rates and transmission risk (without an overall increase in mosquito abundance)^70^.

Many studies aimed at projecting future outbreaks have not been validated on historic or current surveillance data. For USUV, we used a validated model to derive parameter values used in the Next Generation Matrix, thereby improving the reliability of our results. This model has been calibrated to five sources of surveillance data collected in the Netherlands in the period 2016–2022^34^. Additionally, we built on previously published projections of mosquito and bird abundance, which were not only based on temperature scenarios, but also changes in land use and other environmental variables. However, the exact values of R_0_ and R_0_-based indicators should be interpreted with care, especially for WNV. While parameter estimates for USUV were obtained from an USUV transmission model calibrated to the Dutch context^34^, such calibrated parameters were not available for WNV, due to the currently limited transmission in the Netherlands. We therefore relied on published literature for infection parameters and assumed a similarly sized competent host population as compared to USUV. This resulted in a lower estimated R_0_ for WNV compared to USUV, which is in line with lower observed seroprevalence and fewer cases being detected for WNV compared to USUV^15^. Validation of the estimated spatial trends of WNV risk is challenging due to the limited number of seropositive birds to date in which no clear spatial trends are visible^15^. Moreover, due to both viruses only emerging recently, spatial trends in seroprevalence do not solely reflect suitability. For USUV, the estimated higher R_0_ values in the South compared to the North match with reported regional seroprevalence and infection levels also in the years after the initial emergence phase^34^.

A wide range of bird species have been found competent for USUV and WNV transmission. These species differ in their competence and distribution, thereby leading to spatial heterogeneity in transmission risk. Our sensitivity analysis showed that the choice of host species affected the spatial pattern in disease risk, yet not the general trends. This shows that a better understanding of which species form the main competent host population would improve the accuracy of our results, especially regarding spatial heterogeneity. In reality, these spatial patterns are not only affected by distribution of competent hosts, but also of non-competent hosts. Our results suggested that future changes in bird abundance alone do not increase transmission risk. Due to a lack of information on the distribution of all possible *Cx pipiens* hosts, we assumed the total host population to be the same in each location. We also assumed that future changes in the competent bird species were reflective of changes in the total host population and therefore, in future scenarios, kept the overall ratio of competent vs. non-competent hosts equal to the reference scenario. While these assumptions are unlikely to impact the overall differences between scenarios, they do impact local-level projections and predicted effects of future bird abundance. Should the abundance of competent hosts increase more than that of non-competent hosts, transmission risk would increase further. It is also important to note that results are based on the basic reproduction number and therefore the metrics presented do not account for immunity in the population. Given the ongoing circulation of both viruses in the Netherlands^33^, immunity will play an important role in shaping future transmission. Other possible directions for future extensions include exploring the impact of future scenarios on diapause initiation and duration as well as on bird movement patterns. It has been shown that mosquitoes can adapt to changing climate through evolution, which could affect their transmission potential in future scenarios^71,72^. Moreover, we compared scenarios based on the mean generation interval, but more detailed approaches for calculating the distribution of generation intervals, such as presented in^65,73^, could be adapted to USUV and WNV to study this in greater detail.

Our results indicate that WNV and USUV outbreaks are expected to grow more rapidly in the future. This means that timely surveillance becomes more challenging and the window of opportunity for response becomes smaller. Should human vaccines become available in the future, these will have to be rolled out faster if a reactive vaccination strategy is followed. The expected increase in transmission potential between mosquitoes and birds is likely to also lead to more spillover cases to other animals, including humans, due to the generalist feeding behaviour of *Cx pipiens* mosquitoes^74^. It also creates more opportunities for evolution to occur, possibly further facilitating transmission. Future scenarios do not only differ in their projected transmission risk, but also in their ability to anticipate and respond to outbreaks^28^. The smallest increase in transmission risk was observed in SSP1. Health services in this scenario are also well-prepared for outbreaks and have effective early-warning systems^75^. People are generally healthy and adhere to public health messages. This contrasts with SSP3 where transmission risk increased the most. The impact of larger outbreaks in this scenario is further exacerbated by a lack of a coordinated response, a less healthy population, and lower adherence to public health messages. The predicted increase in future transmission risk is likely to also apply to other arboviruses, including those transmitted by ticks and midges^76,77^. For example, optimal temperature for transmission of Rift Valley Fever is around 26 °C^45^, which is above current mean daily temperatures. Exotic mosquito species could become established in more parts of Europe under changing ecological conditions, including *Ae aegypti* and *Ae albopictus*^10,78^, known vectors for dengue and chikungunya viruses. Combined with high levels of travel and trade, as is predicted especially for SSP4 and SSP5, this leads to a higher risk of newly emerging arboviruses.

## Conclusion

Across all future scenarios, USUV and WNV outbreaks are expected to become larger, last longer, and spread more rapidly, especially in the southern and eastern parts of the Netherlands. This is mostly driven by increased temperatures, suggesting that climate mitigation measures are an effective strategy to reduce outbreak risk and impact. These findings highlight the need for increased preparedness for arbovirus outbreaks in the future. The development and implementation of surveillance and response activities, including potential vaccines or treatment options, is important to mitigate future arbovirus risks for both humans and animals.

## Data Availability

All data used in this study have been published before. Future climate data was taken from scenarios developed by the Dutch Meteorological Organisation for the period 2036-2065 (van Dorland et al., KNMI National Climate Scenarios 2023 for the Netherlands. De Bilt: 2024.). Data for these scenarios, including the reference scenario, is available from the KNMI website (https://klimaatscenarios-data.knmi.nl/downloads). *Cx pipiens* abundance estimates have been published in Krol et al. 2024 (https://doi.org/10.21203/RS.3.RS-5298493/V1) and are available upon request from the corresponding author. Bird abundance estimates have been published in Dellar et al. 2024 (https://doi.org/10.1007/s10393-025-01727-9). The explanatory variables, model outputs, code, and supplementary information for the bird abundance estimates can be accessed via the Dryad repository: https://doi.org/10.5061/dryad.r2280gbmc. The bird count data is not publicly available but can be requested from the Dutch Centre for Field Ornithology (Sovon: sovon.nl) or Vigie-Nature (https://www.vigienature.fr/fr/suivi-temporel-desoiseaux-communs-stoc). A summary of the bird data is available via the above Dryad link. The land use maps associated with each scenario have been published in Dellar et al. 2024 (https://doi.org/10.1038/s41597-024-04059-5). For land use maps analysis, all information and results is available via the Dryad repository: https://doi.org/10.5061/dryad.sj3tx96bs.

## Declarations

### Code availability

All code is available via GitHub at https://github.com/Mariken95/VBD-scenarios.
